# Supplementary material for: The Mitochondria‐Targeted Peptide Therapeutic Elamipretide Improves Cardiac and Skeletal Muscle Function During Aging Without Detectable Changes in Tissue Epigenetic or Transcriptomic Age
Source: Aging Cell. 2025 Mar 13;24(6):e70026. doi: 10.1111/acel.70026 (PMC12151887; doi:10.1111/acel.70026)
Supplement: Supplementary file 1 — Appendix S1. [file ACEL-24-e70026-s003.zip › Extended Data Figure captions.docx]

**Extended Data** Figure 1**: Similar components of the 31-point frailty index are elevated in old control- and ELAM-treated mice.** Heatmap of the average value of each component of the frailty index in old control- (Con) and ELAM-treated male (M) and female (F) mice (*n* = 11–12) at ages of 24, 25, and 26 months old. Only mice that survived to the study endpoint were included in the analysis.

**Extended Data** Figure 2**: Aging and elamipretide treatment analyzed by echocardiography strain analysis.** *Baseline echocardiography strain analysis of parasternal long axis images of the LV of young (Yng) and old mice for A. End diastolic volume (EDV), B. End systolic volume (ESV), C. Stroke Volume (SV), D. Fractional shortening (FS), E. Cardiac output (CO), F. End diastolic left ventricular mass (EDLVM), G. End systolic left ventricular mass (ESLVM), and H. Heart rate. Δ (Post-pre) echocardiography strain analysis of parasternal long axis images of the LV of aged control (Con) and ELAM-treated mice for I. EDV, J. ESV, K. SV, L. FS, M. CO, N. EDLVM, O. ESLV), and P. Heart rate.* 4-5-month-old (young) female and male (*n* = 9–10) and 23-24-month-old (old) female (*n* = 23–24) and male were compared for each measurement at baseline. ELAM treatment effects were compared in control and ELAM-treated old mice (*n* = 11–12) using Δ measurements. Only mice that survived to study endpoint were included in the analysis. Statistical significance was determined by two-way ANOVA with Tukey's post hoc test. Significant ANOVA factors written in text with selected Tukey's post hoc test comparisons on graphs. Error bars represent sample means ± standard deviations.

**Extended Data** Figure 3**: Systolic function in aging and elamipretide treatment analyzed by conventional echocardiography.** *Baseline conventional echocardiography systolic function quantified from the short axis M-mode images of the LV of young (Yng) and old mice for A. End systolic diameter (ESD), B. End diastolic diameter (EDD), C. ESV, D. EDV, E. SV, F. Ejection fraction (EF), G. FS, and H. CO. Δ (Post-pre) conventional echocardiography systolic function quantified from the short axis M-mode images of the LV of aged control (Con) and ELAM-treated mice for I. ESV, J. EDD, K. ESV, L. EDV, M. SV, N. EF, O. FS, and P. CO. 4- to*-month-old (young) female and male (*n* = 9–10) and 23- to4-month-old (old) female (*n* = 23–24) and male were compared for each measurement at baseline. ELAM treatment effects were compared in control and ELAM-treated old mice (*n* = 11–12) using Δ measurements. Only mice that survived to the study endpoint were included in the analysis. Statistical significance was determined by two-way ANOVA with Tukey's post hoc test. Significant ANOVA factors written in text with selected Tukey's post hoc test comparisons on graphs. Error bars represent sample means ± standard deviations.

**Extended Data** Figure 4**: Diastolic function in aging and elamipretide treatment analyzed by conventional echocardiography.** *Baseline conventional echocardiography diastolic function quantified from 4-chamber tissue doppler and pulse wave doppler images of the LV of young (Yng) and old mice for A. Mitral valve (MV) E wave, B. MV A wave, C. E' wave, D. A' wave, E. MV E/E**', F. MV E/A, G. E**'/A**', and H. A**'/E**'. Δ (Post-pre) conventional echocardiography diastolic function quantified from 4-chamber tissue doppler and pulse wave doppler images of the LV of aged control (Con) and ELAM treated mice for I. MV E wave, J. MV A wave, K. E' wave, L. A' wave, M. MV E/E**', N. MV E/A, O. E/A**', P. A**'/E**'.* 4-5-month-old (young) female and male (n = 9–10) and 23-24-month-old (old) female (n = 23–24) and male were compared for each measurement at baseline. ELAM treatment effects were compared in control and ELAM-treated old mice (n = 11–12) using Δ measurements. Only mice that survived to study endpoint were included in the analysis. Statistical significance was determined by two-way ANOVA with Tukey's post hoc test. Significant ANOVA factors are written in text with selected Tukey's post hoc test comparisons on graphs. Error bars represent sample means ± standard deviations.

**Extended Data Figure 5: Cardiac hypertrophy in aging and elamipretide treatment analyzed by conventional echocardiography.** *Baseline conventional echocardiography cardiac hypertrophy from the short axis M-mode images of the LV of young (Yng) and old mice for A. LV Mass, B. LV Mass Cor (corrected), C. Left ventricular anterior wall thickness in systole (LVAW;s), D. Left ventricular anterior wall thickness in diastole (LVAW;d), E. Left ventricular posterior wall thickness in systole (LVPW;s), F. Left ventricular posterior wall thickness in diastole (LVPW;d). At study endpoint, the heart was collected and weighed for control young and old mice for G. Heart mass, and H. Heart mass normalized to tibia length. Δ (Post-pre) conventional echocardiography cardiac hypertrophy from the short axis M-mode images of the LV of aged control (Con) and ELAM-treated mice for I. LV Mass, J. LV Mass Cor, K. LVAW;s, L. LVAW;d, M. LVPW;s, N. LVPW;d**. At study endpoint, the heart was collected and weighed for control and ELAM-treated old mice for G. Heart mass, and H. Heart mass normalized to tibia length.* 5-month-old (young) female and male (*n* = 9–10) and 23–24-month-old (old) female (*n* = 23–24) and male were compared for each measurement at baseline. ELAM treatment effects were compared in control and ELAM-treated old mice (*n* = 11–12) using Δ measurements. Only mice that survived to the study endpoint were included in the analysis. Heart masses were collected at the study endpoint from 7-month (young) and 26-month (old) male and female control and ELAM-treated mice. Statistical significance was determined by two-way ANOVA with Tukey's post hoc test. Significant ANOVA factors written in text with selected Tukey's post hoc test comparisons on graphs. Error bars represent sample means ± standard deviations.

**Extended Data Figure 6: In vivo muscle force-frequency in aging and elamipretide treatment.** *At study endpoint, the gastrocnemius muscles were collected, weighed, and normalized to tibia length for A. Control young and old mice, and B. Control and ELAM-treated old mice. C. Baseline* in vivo *muscle force normalized to body mass across a range of stimulation frequencies in young and old mice. D. Baseline maximum* in vivo *muscle force normalized to body mass of young and old mice. E. Δ (Post-pre)* in vivo *muscle force normalized to body mass across a range of stimulation frequencies at endpoint for old mice. F. Δ maximum* in vivo *muscle force normalized to body mass at endpoint for old mice. G. Baseline maximum rate of muscle contraction across a range of stimulation frequencies in young and old mice. H. Δ maximum rate of muscle contraction across a range of stimulation frequencies at endpoint for old control and ELAM mice. I. Baseline maximum rate of muscle relaxation across a range of stimulation frequencies in young and old mice. J. Δ maximum rate of muscle relaxation across a range of stimulation frequencies at endpoint for old control and ELAM-treated mice.* 5-month-old (young) female and male (*n* = 9–10) and 23–24-month-old (old) female (*n* = 23–24) and male were compared for each measurement at baseline. ELAM treatment effects were compared in control and ELAM-treated old mice (*n* = 11–12) using Δ measurements. Only mice that survived to study endpoint were included in the analysis. Gastrocnemius muscle masses were collected at study endpoint from 7-month (young) and 26-month (old) male and female control and ELAM-treated mice. Statistical significance was determined by two-way ANOVA with Tukey's post hoc test except for comparisons that include stimulation frequency as a factor, which were analyzed by three-way ANOVA. Significant ANOVA factors written in text with selected Tukey's post hoc test comparisons on graphs. Error bars represent sample means ± standard deviations.

**Extended Data Figure 7: In vivo muscle fatigue contraction and relaxation in aging and elamipretide treatment.** *A. Baseline maximum rate of muscle contraction across 120 fatiguing stimulations in young and old mice. B. Δ (Post-pre) maximum rate of muscle contraction across 120 fatiguing stimulations at endpoint for old control and ELAM-treated mice. C. Baseline maximum rate of muscle relaxation across 120 fatiguing stimulations in young and old mice. D. Δ maximum rate of muscle relaxation across 120 fatiguing stimulations at endpoint for old control and ELAM-treated mice.* 5-month-old (young) female and male (n = 9–10) and 23–24-month-old (old) female (n = 23–24) and male were compared for each measurement at baseline. ELAM treatment effects were compared in control and ELAM-treated old mice (n = 11–12) using Δ measurements. Only mice that survived to the study endpoint were included in the analysis. Statistical significance was determined by three-way ANOVA with Tukey's post hoc test. Significant ANOVA factors written in text with selected Tukey's post hoc test comparisons on graphs. Error bars omitted for clarity.

**Extended Data Figure 8: Effect of 2-months ELAM treatment on aging-related changes in gene expression.**

*A. Correlation analysis of gene expression changes induced by ELAM treatment at the level of individual genes (left) and enriched pathways (right).* Spearman correlation between normalized enrichment scores (NES) from gene set enrichment analysis (GSEA) performed for signatures of ELAM treatment (blue), lifespan-extending interventions (green), and mammalian aging and mortality (red). Adjusted p-value < 0.1, *adjusted p-value < 0.05, **adjusted p-value < 0.01, ***adjusted p-value < 0.001. *B. Modular transcriptomic clock analysis.* Normalized difference in tAge between control and ELAM-treated mice estimated with individual module-specific transcriptomic clocks of chronological age and mortality. Negative (blue) and positive (red) values represent reduced and elevated tAge in ELAM-treated animals, respectively. No statistically significant differences (Benjamini–Hochberg adjusted p-value < 0.05) were observed for any modular clocks. *n* = 4–5 animals per group.

**Extended Data Figure 9: Network analysis of downregulated GObp terms following 2-months ELAM treatment in old mouse hearts.**

*A. Females. B. Males.* Gene Ontology biological process (GObp) terms that were significantly upregulated (False Discovery Rate < 0.05) were first consolidated using REVIGO (Supek et al., [2011](#B83)) before visualization using Cytoscape (Shannon et al., [2003](#B76)). *n* = 4–5 animals per group.

**Extended Data Figure 10: Pearson correlation and post-treatment** **analyses.**

*A.* *Correlation of LV mass with tAge.* Data points correspond to LV mass (post-treatment) and tAge (relative) of hearts from young and old male and female mice (both treatments). *B. Correlation of tAge with GLS.* Data points correspond to ΔGLS (Post-pre) and tAge (relative) of hearts from old male and female mice (both treatments). *C. Correlation of DNAmAge with tAge.* Data points correspond to DNAmAge (PanTissue) and tAge (relative) of hearts from young and old male and female mice (both treatments). *D. Effects of ELAM on post-treatment frailty index in old mice.* Data is only shown for mice whose tissues were subjected to the omics experiments (both sexes combined, *n* = 9–10 mice per group). P-value was determined by unpaired two-tailed *t*-test. *E. Effects of ELAM on post-treatment GLS.* Data is only shown for mice whose tissues were subjected to the omics experiments (n = 4–5 mice per group). P-values were determined by two-way ANOVA and Šidák correction.

**Extended Data Figure 11: Normalized gene expression of CIT targets.**

*A. Hsp70. B. TFAM. C. MGMT.* Benjamini–Hochberg adjusted p-values comparing sex- and age-matched control versus ELAM treated mice are shown in text. n = 4–5 animals per group.
