# Supplementary material for: The Mitochondria‐Targeted Peptide Therapeutic Elamipretide Improves Cardiac and Skeletal Muscle Function During Aging Without Detectable Changes in Tissue Epigenetic or Transcriptomic Age
Source: Aging Cell. 2025 Mar 13;24(6):e70026. doi: 10.1111/acel.70026 (PMC12151887; doi:10.1111/acel.70026)
Supplement: Supplementary file 1 — Appendix S1. [file ACEL-24-e70026-s003.zip › ACEL70026-sup-0001-Extended_Data_Figures RV1.pdf]

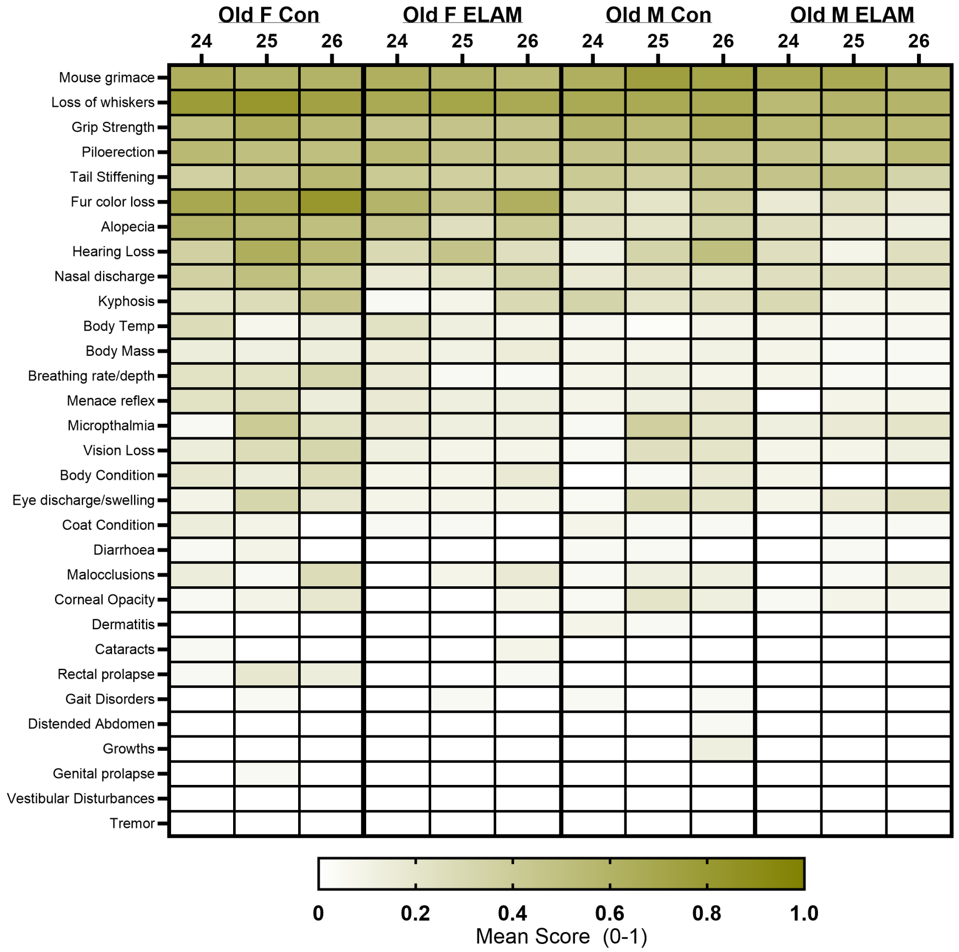

Extended Data Figure 1

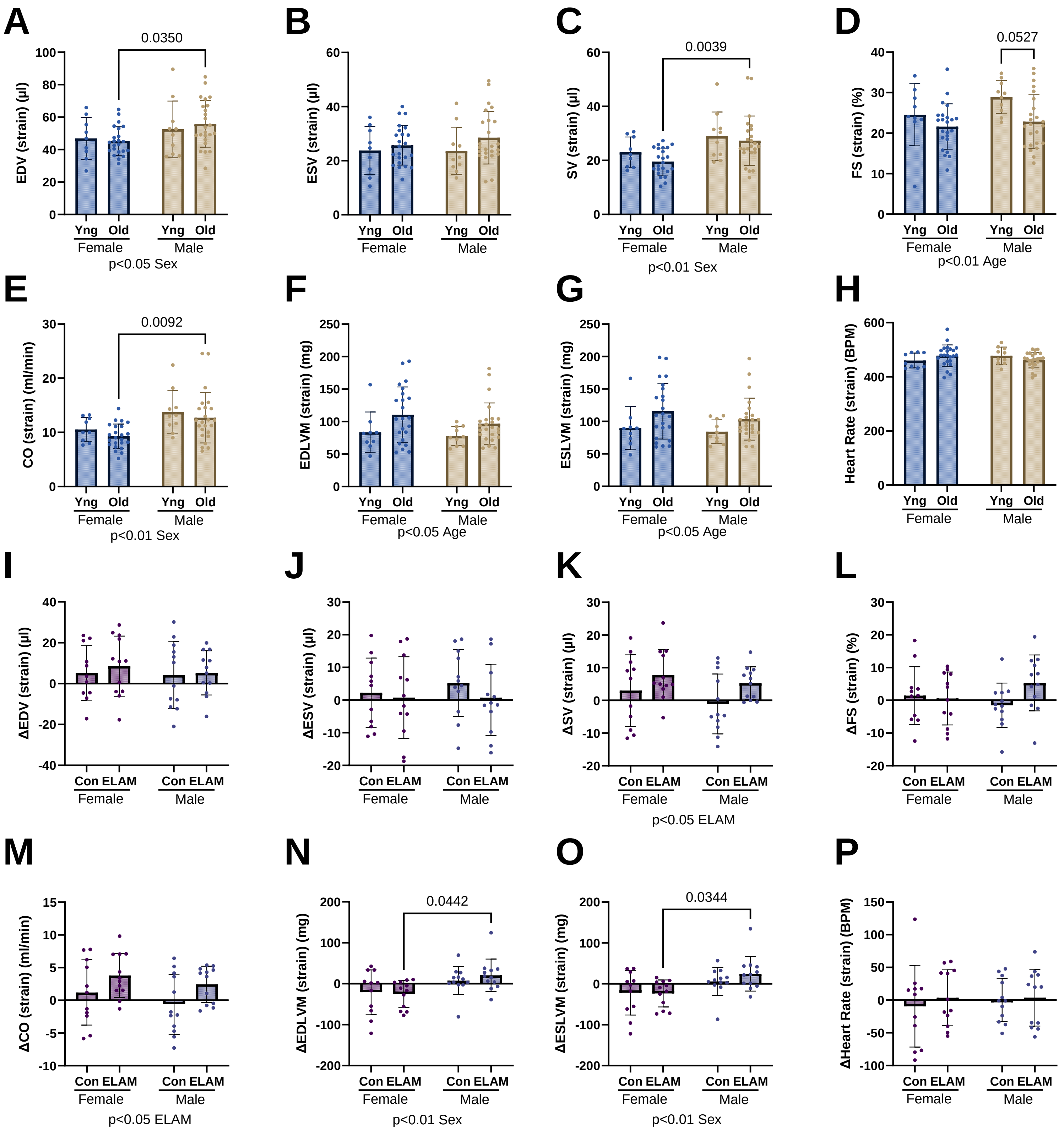

Extended Data Figure 2

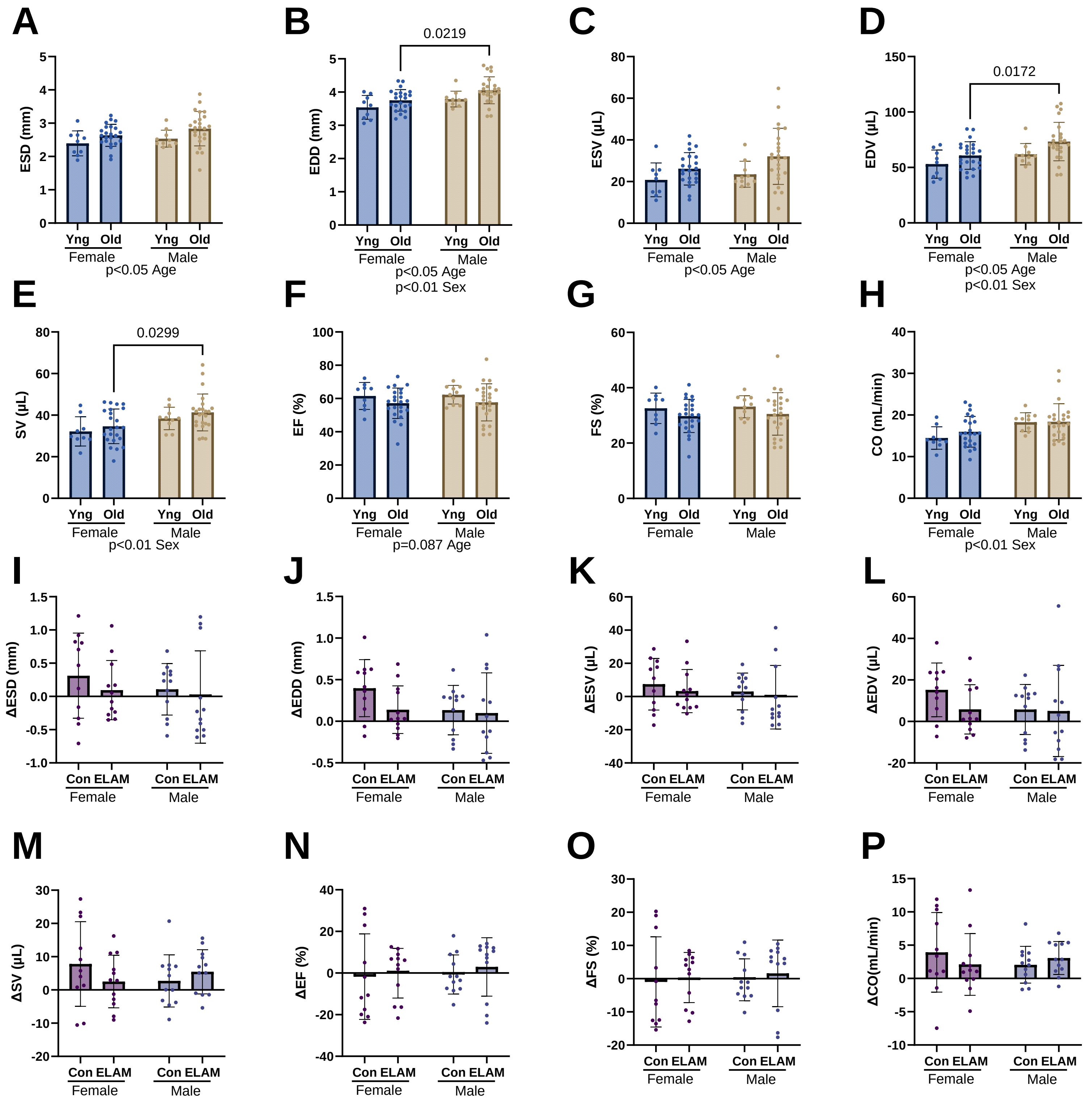

Extended Data Figure 3

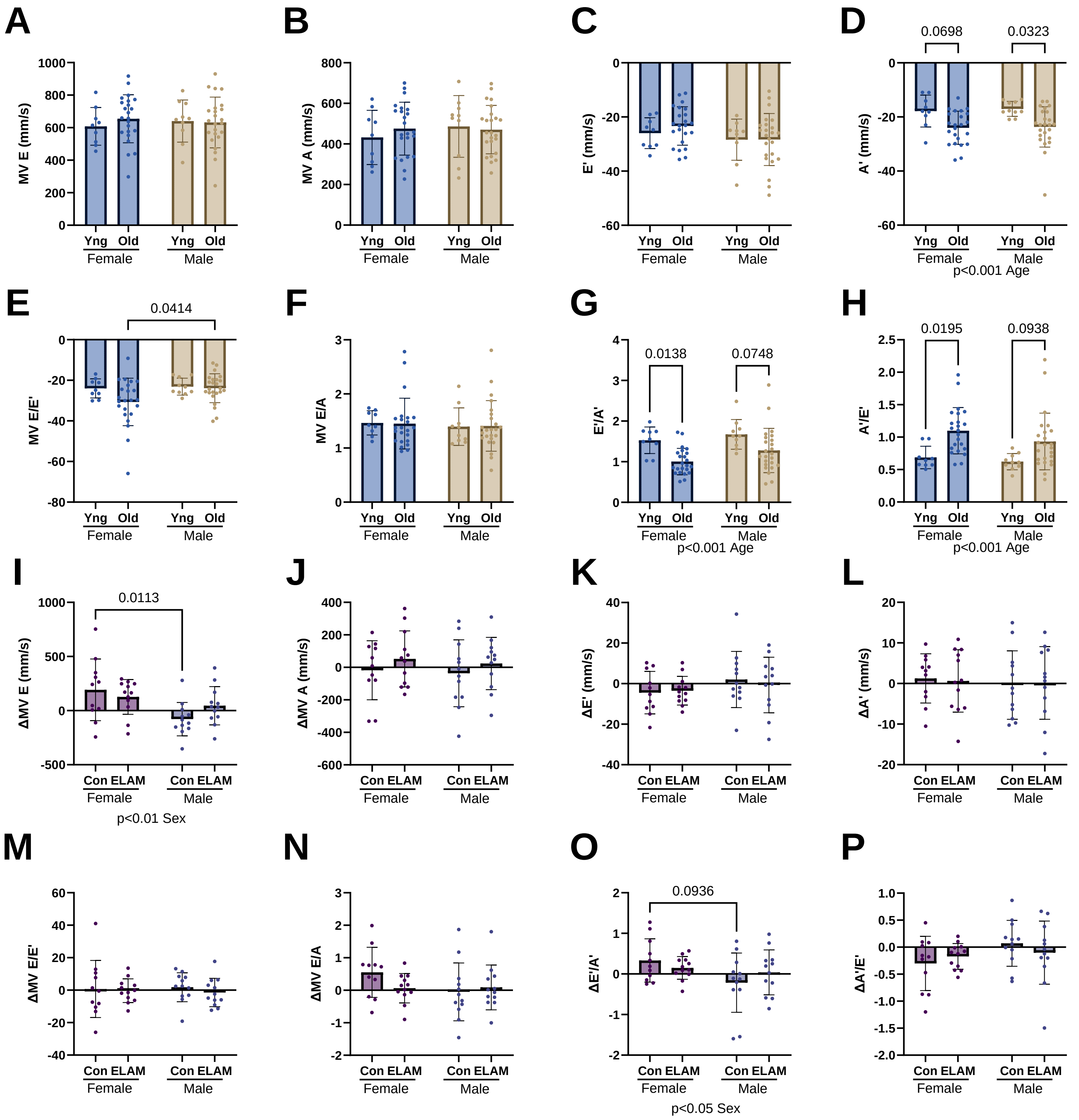

Extended Data Figure 4

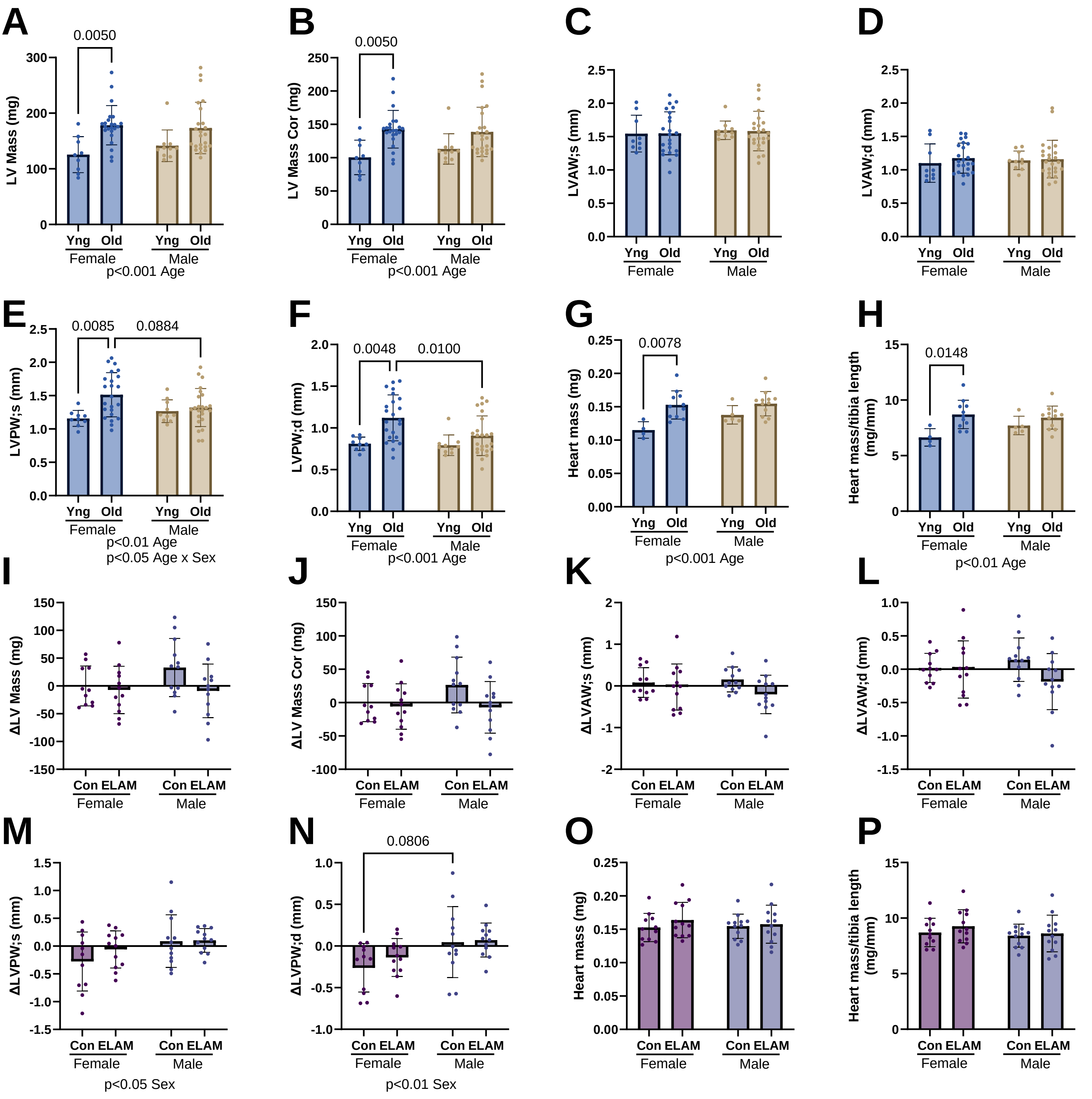

Extended Data Figure 5

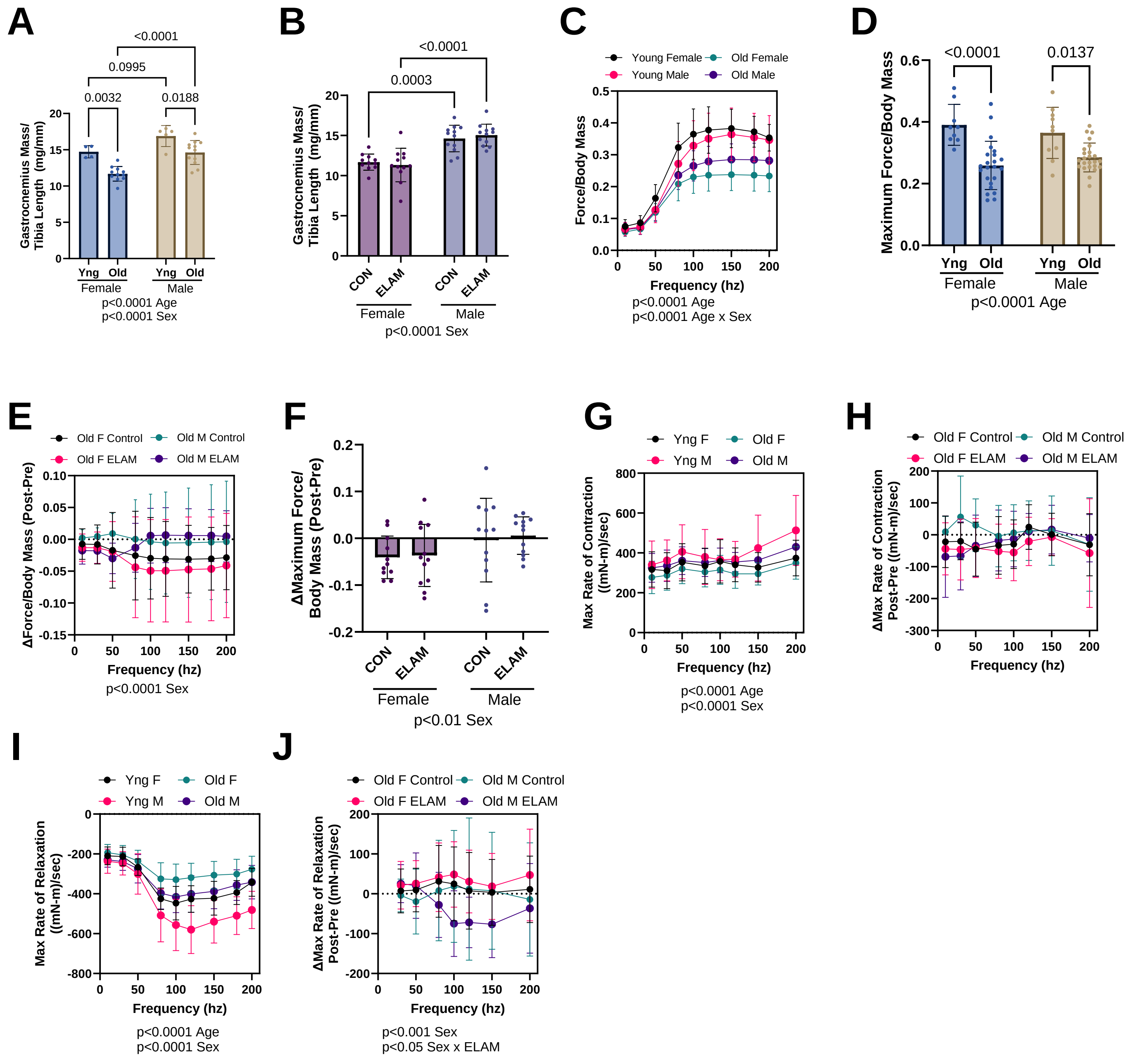

Extended Data Figure 6

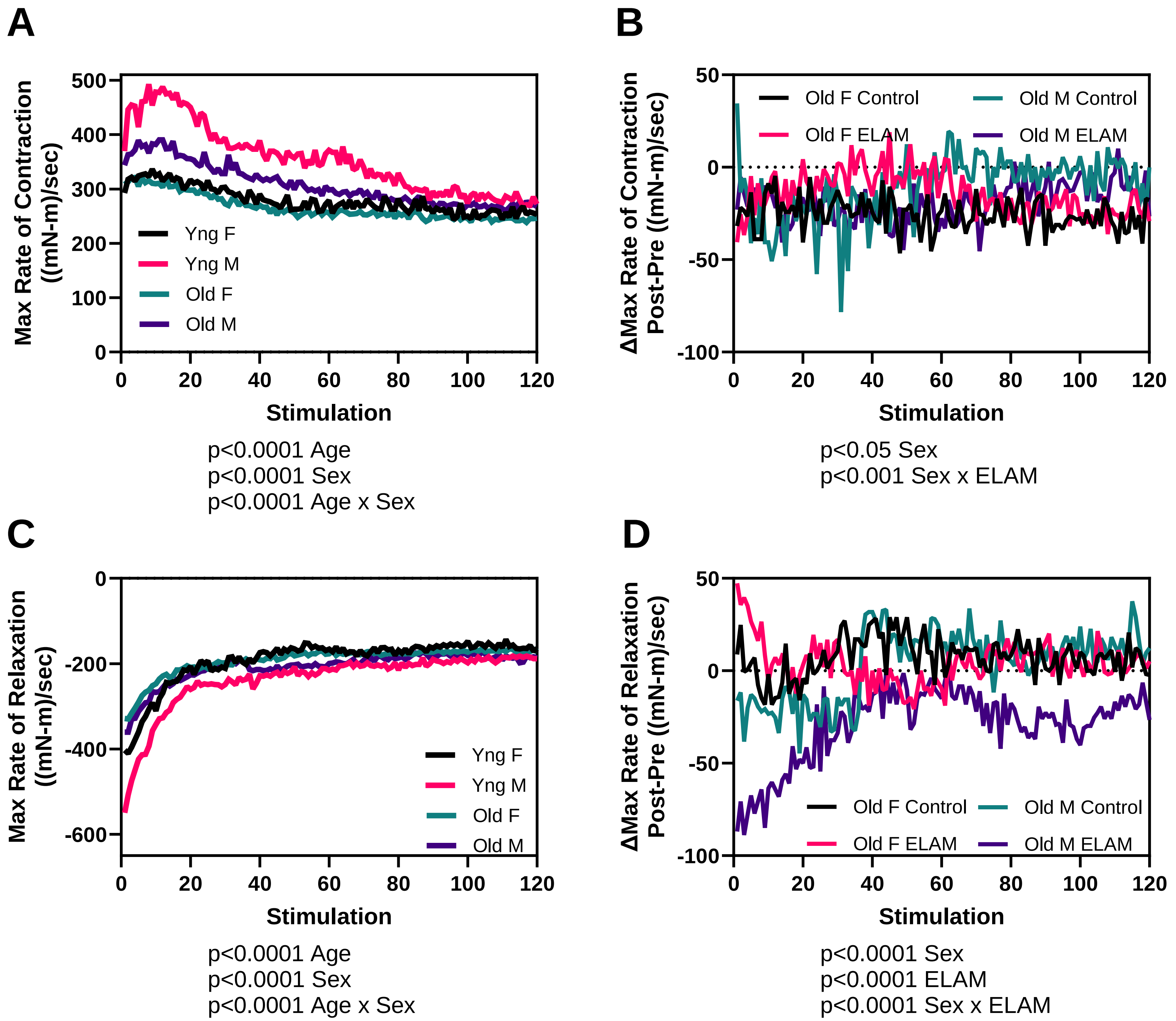

Extended Data Figure 7

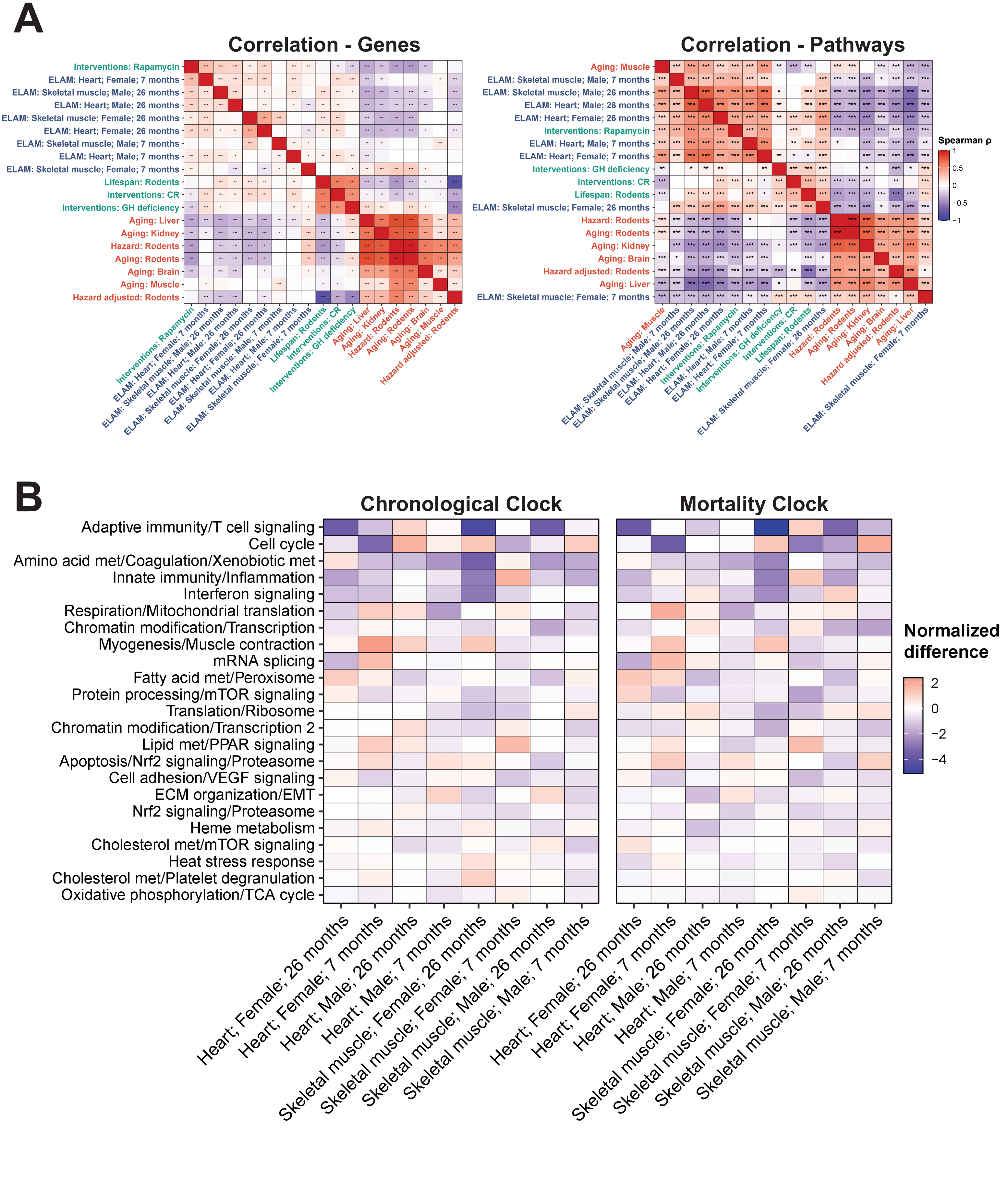

Extended Data Figure 8

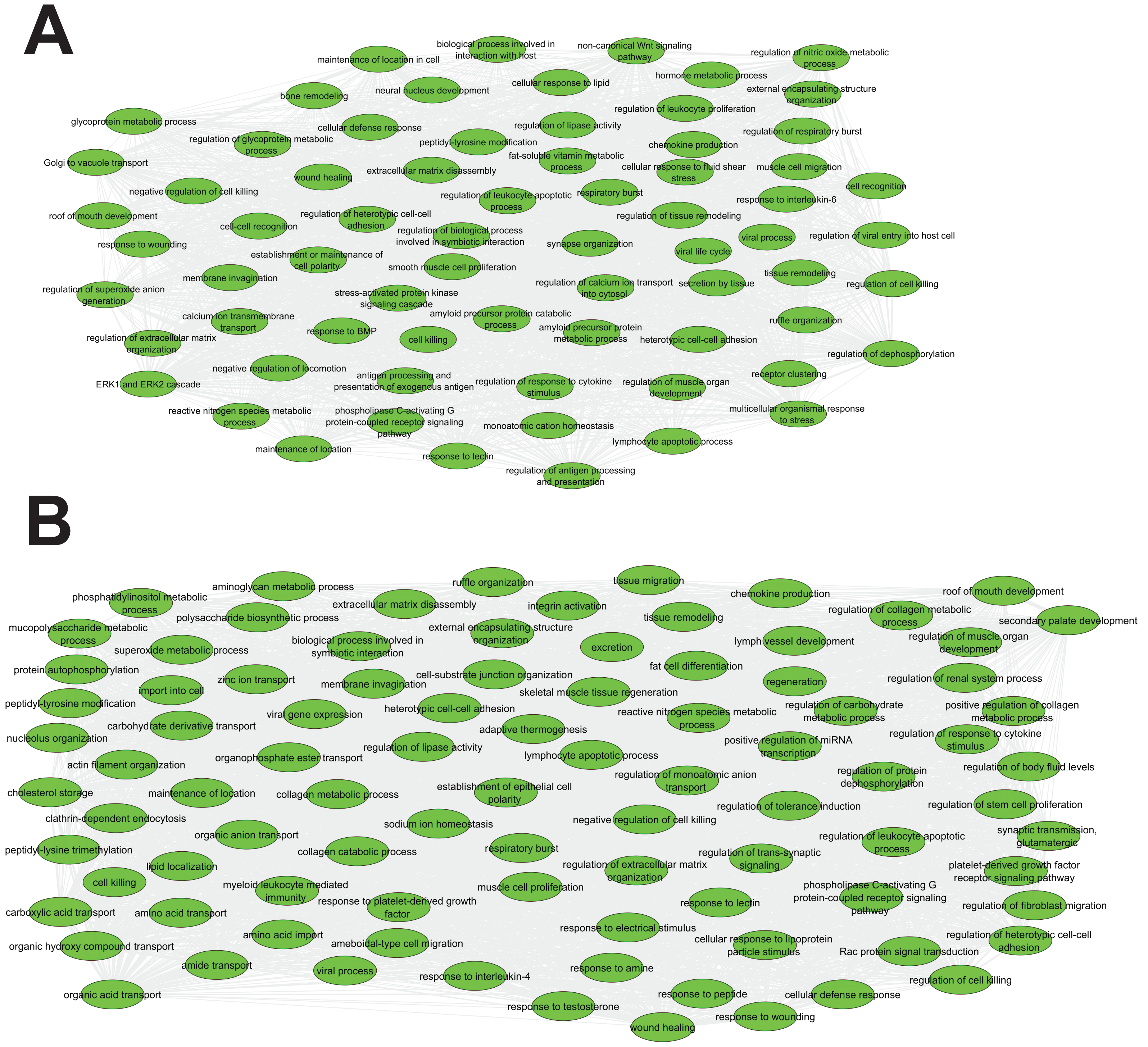

Extended Data Figure 9

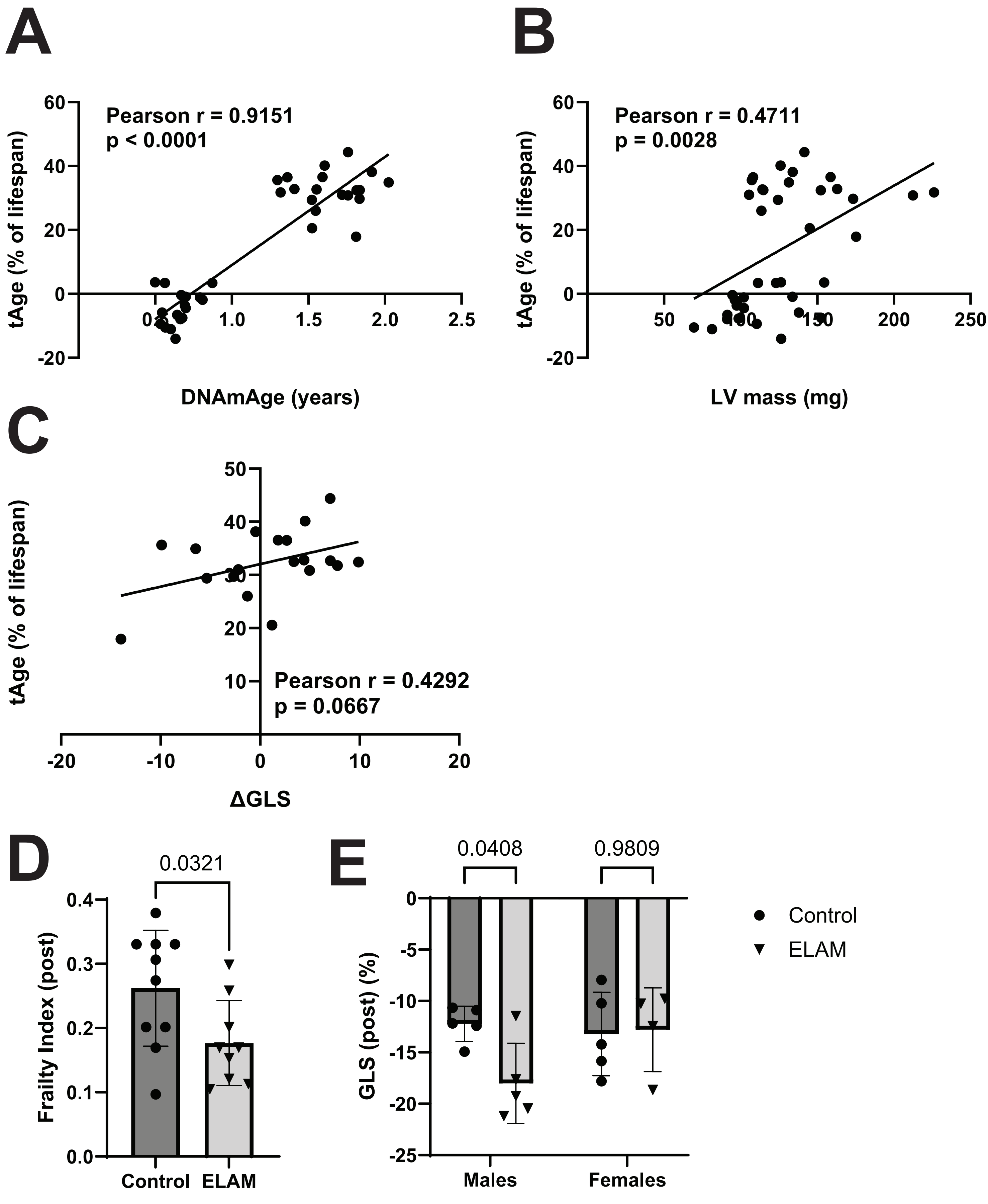

Extended Data Figure 10

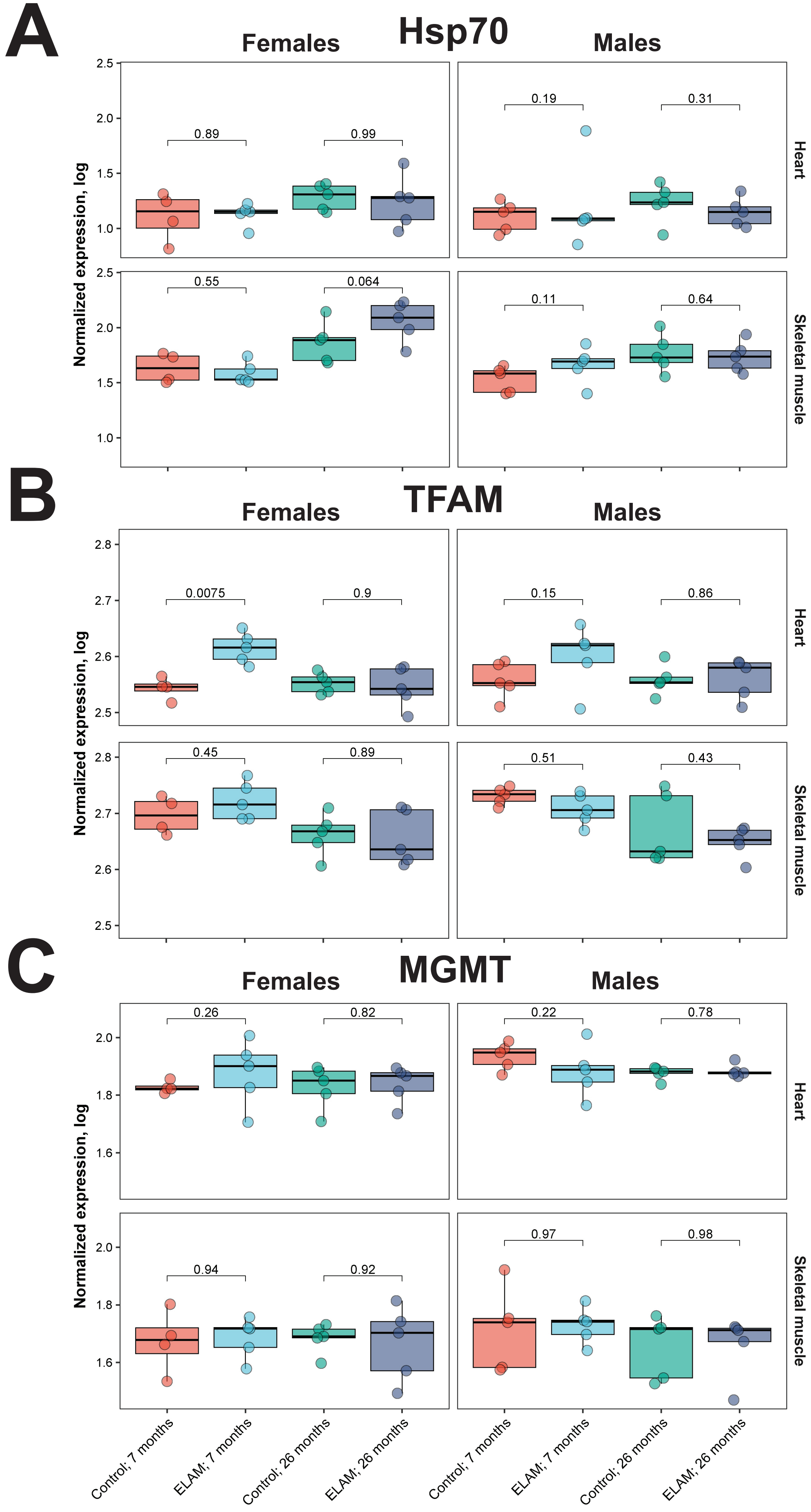

Extended Data Figure 11
